# A study protocol for a multicenter randomized pilot trial of a dyadic, tailored, web-based, psychosocial, and physical activity self-management program (*TEMPO*) for men with prostate cancer and their caregivers

**DOI:** 10.1186/s40814-021-00791-6

**Published:** 2021-03-20

**Authors:** Sylvie D. Lambert, Lindsay R. Duncan, Janet Ellis, John Wellesley Robinson, Carly Sears, Nicole Culos-Reed, Andrew Matthew, Manon De Raad, Jamie Lynn Schaffler, Daniel Santa Mina, Paramita Saha-Chaudhuri, Helen McTaggart-Cowan, Stuart Peacock

**Affiliations:** 1 Ingram School of Nursing, McGill University, Montreal, Quebec Canada; 2 St. Mary’s Research Centre, Montreal, Quebec Canada; 3 Department of Kinesiology and Physical Education, McGill University, Montreal, Quebec Canada; 4 Department of Psychiatry, University of Toronto, Toronto, Canada; 5 Psychosocial Care in Trauma, Sunnybrook Health Sciences Centre, Toronto, Canada; 6 Department of Psychology, University of Calgary, Calgary, Canada; 7 Department of Oncology, University of Calgary, Calgary, Canada; 8 University of Calgary, Calgary, Canada; 9 Health and Exercise Psychology, Faculty of Kinesiology, University of Calgary, Calgary, Canada; 10 Department of Oncology, Cumming School of Medicine, University of Calgary, Calgary, Canada; 11 Psychosocial Resources, Tom Baker Cancer Centre, Cancer Care, Alberta Health Services, Edmonton, Canada; 12 Faculty of Medicine, Department of Surgery, University of Toronto, Toronto, Canada; 13 Department of Psychiatry, University of Toronto, Toronto, Canada; 14 Department of Surgery, Princess Margaret Cancer Centre, Toronto, Canada; 15 Faculty of Kinesiology and Physical Education, University of Toronto, Toronto, Canada; 16 Toronto General Hospital, Toronto, Canada; 17 Department of Mathematics and Statistics, University of Vermont, Burlington, VT USA; 18 Canadian Centre for Applied Research in Cancer Control, Toronto, Canada; 19 Cancer Control Research, BC Cancer, Vancouver, Canada; 20 Faculty of Health Sciences, Simon Fraser University, Burnaby, Canada

**Keywords:** Prostate cancer, Cancer survivorship, Cancer rehabilitation, Self-management, Caregivers, Dyadic intervention

## Abstract

**Background:**

Prostate cancer predisposes patients and caregivers to a wide range of complex physical and psychosocial challenges, and interventions must incorporate a wide range of self-management strategies to help patients and their caregivers effectively cope with cancer challenges. To palliate this need, our team recently developed and evaluated the initial acceptability of a dyadic, Tailored, wEb-based, psychosocial, and physical activity self-Management PrOgram (*TEMPO*). *TEMPO* is a 10-week, interactive, web-based intervention consisting of five modules designed to help dyads manage their physical and psychosocial needs. It aims to teach dyads new self-management strategies and encourages them to increase their physical activity (PA) levels, mainly through walking and strength-based exercises. Initial acceptability evaluation of *TEMPO* revealed high user satisfaction, in addition to having a number of potential benefits for participants. After integrating suggested changes to *TEMPO*, the proposed pilot study aims to further test the acceptability and feasibility of *TEMPO*.

**Methods:**

This study is a multicenter, stratified, parallel, two-group, pilot randomized control trial (RCT), where patient–caregiver dyads are randomized (stratified by anxiety level) to receive (a) *TEMPO* or (b) usual care. Participants (*n* goal = 40) are recruited across Canada at participating cancer centers and through self-referral (e.g., online recruitment). Patient inclusion criteria are (a) having received prostate cancer treatment within the past 2 years or scheduled to receive treatment, (b) identified a primary caregiver willing to participate in the study, and (c) has access to the Internet. Eligible caregivers are those identified by the patient as his primary source of support. Dyads complete a baseline questionnaire (T1) and another one 3 months later (T2) assessing various aspects of physical and emotional functioning (e.g., the Medical Outcomes Study (MOS) 12-item Short Form Health Survey (SF-12), the Hospital Anxiety and Depression Scale (HADS), and the Perceived Stress Scale (PSS)), self-management behaviors (e.g., the Health Education Impact Questionnaire (heiQ)), physical activity (the International Physical Activity Questionnaires (IPAQ) and the Multidimensional Self-efficacy for Exercise Scale (MSES)), and dyadic coping (the Dyadic Coping Inventory (DCI)). Dyads that used *TEMPO* are also asked to participate in a semi-structured exit interview exploring their overall experience with the program.

**Discussion:**

This feasibility analysis will begin to develop the knowledge base on *TEMPO*’s value for men with prostate cancer and their caregivers to inform a larger trial.

**Trial registration:**

NCT04304196

**Supplementary Information:**

The online version contains supplementary material available at 10.1186/s40814-021-00791-6.

## Introduction

### Background

Prostate cancer management presents patients and their caregivers with a myriad of complex physical and psychosocial challenges [1–3]. Informal caregivers (most often patients’ partners) help to alleviate the demands on the healthcare system [4], and contribute positively to patients’ illness adjustment [2, 3]. However, caregivers’ extensive support often results in high levels of physical, emotional, and social burden. A recent longitudinal analysis highlighted that 53% of caregivers report chronically poor physical functioning, and an additional 17% experience a steep decline in physical health over the first 5 years of providing care for a newly diagnosed person with cancer [5]. Likewise, the incidence of clinically significant levels of anxiety and depression among prostate cancer caregivers supersedes those of the patient [6], and these symptoms may persist for up to 5 years [7]. As such**,** 16 to 68% of caregivers report needing more support [3], particularly in curtailing the emotional and psychological impacts of cancer, and help looking after their own health.

A number of interventions have been developed to address caregivers’ unmet supportive care needs and enhance their quality of life (QOL) [8–10]. Most of these interventions are grounded in the principles of psycho-education and self-management to effectively address caregivers’ psychosocial and emotional needs [8, 9, 11], but fail to incorporate strategies to enhance caregivers’ physical well-being. As such, emerging literature [12, 13] has focused on incorporating regular physical activity (PA) into self-management interventions as a strategy to curtail the negative physical and emotional sequela associated with cancer caregiving. In line with this, in a survey on cancer caregivers’ preferences for stress-management strategies, nearly 75% of respondents stated that they were most interested in programs with an exercise component to reduce burden [12].

In recent years, two systematic reviews [13, 14] have collectively identified 17 PA interventions for family caregivers. Findings from these reviews provide preliminary evidence for the efficacy of PA interventions on caregivers’ psychosocial outcomes, notably in reducing distress, and increasing well-being, QOL, and self-efficacy [13]. However, only three [15–17] of the 17 interventions were developed for cancer caregivers. Martin and colleagues [16] designed a 6-week, group-based yoga intervention for caregivers of cancer survivors, whereas the other two interventions utilized a dyadic approach to engage breast [15] or lung [17] cancer patients and their partners in a 6-week low-intensity walking or yoga program, respectively. These dyadic PA interventions were found to lead to a significant decrease in anxiety and depressive symptoms for both members of the dyad, in addition to providing individual benefits (e.g., decreased sleep disturbances among caregivers) [15, 17]. These systematic reviews [13, 14] identified a need for more rigorous trials to support this approach in improving cancer caregivers’ outcomes. This is in line with studies suggesting that coordinating coping effort within the patient–caregiver dyad, such as engaging in PA together, can contribute significantly to both the patients’ and caregivers’ well-being [18, 19]. Dyadic PA interventions conducted with non-cancer caregivers have also been found to be efficacious [13].

Since the aforementioned caregiver PA intervention reviews have been published [13, 14], the RECHARGE dyadic PA intervention for cancer dyads has been developed and aims to capitalize on the opportunity to enhance both patient and caregiver outcomes [20]. RECHARGE is a 12-week exercise intervention aimed at caregivers providing physical or psychological support to a patient with any type of cancer. It is a structured healthcare provider (HCP)-facilitated intervention consisting of: two weekly group-based resistance exercise sessions, 7 group-based education sessions, and individual weekly aerobic exercise sessions [20]. Although RECHARGE has also been found to be efficacious at improving a number of psychosocial outcomes among dyads [20], the literature suggests that structured HCP-driven interventions may not be sustainable in the long run due to their high cost and lack of flexibility [11]. As such, there is a need to find alternate delivery formats (e.g., self-directed or home-based formats) that provide ongoing instructions and support in a way that is cost-effective and that offer participants the flexibility to choose when and where to engage in the program [21, 22].

Two dyadic cancer interventions for patients and caregivers (FOCUS [23] and CARES [24]) exist in a self-administered web-based format; however, neither incorporates PA self-management strategies. Nonetheless, these studies provide evidence that this approach to program delivery is efficacious in reducing emotional distress and improving QOL and self-efficacy [23, 24]. A recent systematic review [25] of online exercise-based interventions for breast cancer patients (but not their caregivers) found that participant adherence to these programs was generally high. Moreover, participants rated the interventions as acceptable and beneficial, particularly if they assessed them to offer tailored content in a time- and cost-effective manner [25]. Taken together, these studies [20, 23–25] have laid important groundwork for the development of the first dyadic, Tailored, wEb-based, psychosocial and PA self-Management PrOgram (*TEMPO*) for men with prostate cancer and their caregivers.

Our team recently concluded a qualitative study evaluating the acceptability and usefulness of *TEMPO* to facilitate patient–caregiver dyads’ access to QOL-enhancing support and information, tailored to their needs [26]. The findings of this study emphasized that cancer patients and their caregiving partners were satisfied with both *TEMPO*’s dyadic approach (e.g., joint goal setting to integrate self-management skills to cancer care) and the extensive informal support it provided. A number of preliminary benefits were described, notably in the domains of physical well-being (e.g., increased PA and improved overall health), emotional support (e.g., reduced anxiety and improved stress management), psychosocial support (e.g., improved communication, increased teamwork, supported goal fulfillment), and informational support (e.g., increased knowledge and repertoire of self-management skills). Following this initial acceptability study, changes were made to *TEMPO*, including adjustments to imagery and language, improvements to navigation and flow, and finalizing the French version.

### Aims and objectives

The primary aim of this pilot trial is to further test the acceptability and feasibility of *TEMPO*. A secondary aim is to examine the clinical significance of *TEMPO*.

The objectives are to

1. Examine the acceptability of *TEMPO*, including satisfaction, adherence, perceived usefulness, and attrition [27]
2. Examine feasibility defined as rates of recruitment, retention, and questionnaire completion [27]
3. Estimate the clinical significance on anxiety and QOL (primary outcomes), as well as depression, self-management skills, PA, self-efficacy, and appraisal (secondary outcomes)

### Pilot outcomes justifying a larger trial

As a pilot is not suitable for hypothesis testing [28], the focus is on pilot outcomes justifying a larger trial. Given the lack of published benchmarks for feasibility and acceptability, we aim to introduce rigor by nominating the following values:

- The pilot procedures will be feasible if (a) eight dyads/month are recruited across sites, (b) refusal rate does not exceed 45%, (c) missing data are less than 10% [29], and (d) protocol infringements are amenable to change.
- *TEMPO* will be acceptable if (a) attrition in the intervention group does not exceed 25% [30, 31], (b) 75% of dyads adhere to the *TEMPO* modules, and (c) high system usability is reported.
- An appropriate measure of clinical significance for a pilot is the effect size (ES) [CONSORT for pilots [28]]. A clinically (not statistically) meaningful finding is defined as an ES > 0.2 [32] for the primary outcomes (QOL and anxiety) of interest at 3 months post-baseline compared with those in the control group. As a secondary assessment of clinical significance is that 25% of participants improve on the primary outcomes by at the least the Minimal Clinically Important Difference (MCID).

## Methods

### Design

The proposed study is a multicenter, stratified, 1:1, parallel, two-group pilot randomized control trial, whereby patient–caregiver dyads are randomized to receive (a) *TEMPO* or (b) usual care. At the conclusion of the pilot study, semi-structured exit interviews are conducted. The study design was guided by the CONSORT checklist [33] and its adaptation to pilot trials [28]. The reporting of this protocol is according to the SPIRIT guidelines [34]. Supplementary Material S1 includes both the CONSORT and SPIRIT checklists.

### Methodological framework

The *Complex Interventions Framework* [35] describes four steps in the development and evaluation of complex interventions: (a) development, (b) feasibility/pilot testing, (c) evaluation, and (d) implementation. This study focuses on (b).

### Sample and setting

A convenience sample of patients and their caregivers is being recruited (as a dyad) from the McGill University Health Centre (MUHC); St. Mary’s Hospital, an installation of the Montréal West Island Integrated University Health and Social Services Centre (SMHC); Tom Baker Cancer Centre (TBCC); Vancouver General Hospital (VGH); and Sunnybrook Health Sciences Centre (SHSC). Recruitment was initiated on April 2020 and concluded in February 2021. Patient inclusion criteria are (a) having received prostate cancer treatment (surgery, chemotherapy, radiation therapy, hormone therapy, and/or brachytherapy) within the past 2 years or scheduled to receive listed treatments, (b) identified a primary caregiver willing to participate in the study, and (c) has access to the internet.

Eligible caregivers are those identified by the patient as his primary source of support regardless of the type of support provided (emotional support, help with activities of daily living, etc.) and their relationship to the patient. Patients/dyads in the acute survivorship phase (within 2 years of active cancer treatments) are targeted, as this corresponds with a period of high distress for caregivers whereby support is needed to prevent chronic distress [36]. Further, caregivers of men with localized or advanced cancer, and spousal or family caregivers are primary targets for inclusion in the present study, as these caregiver subgroups have all been found to benefit from similar self-management interventions [37–39]. Caregivers who were diagnosed with cancer in the previous year, or who are currently receiving treatment for cancer are excluded. Patients and caregivers both need to be able to understand English or French due to the availability of the *TEMPO* platform in these languages.

### Sample size

Based on other studies [30, 40] and our data from the initial acceptability of *TEMPO* [26], it is assumed that 40% of dyads approached will be ineligible, 40% will refuse participation, 15% will not return their baseline survey, and 25% will be lost to follow-up. Accordingly, 260 dyads will need to be approached to reach the target sample size 40 dyads (20 per group) at the 3-month follow-up [41].

### Recruitment procedures

#### Clinic-based recruitment

Across participating sites, local research assistants (RAs) are calling patients who have previously consented to being contacted about research to introduce the study using information from the study brochure. If the individual is interested, the RA confirms that both the patient and caregiver are eligible. When possible (due to the current restrictions due to COVID-19), RAs work with clinicians at participating sites to identify potentially eligible individuals. With the clinicians’ permission, RAs approach eligible patients either in person or by telephone following their appointment to introduce the study and arrange a time when the screening checklist can be completed in a confidential manner.

#### Community-based recruitment

Dyads are also recruited through self-referral by inviting relevant community organizations across Canada (e.g., Prostate Cancer Canada) to share the study brochure or information from the study brochure with their members, either by posting the information on their social media platform, website, or by circulating it by email. Individuals who are interested are invited to contact the central RA in Montreal using a toll-free number. The RA then answers questions about the study and, if the individual is interested, proceeds with confirmation of eligibility for both the patient and caregiver.

All eligible dyads are invited to complete an online consent form (each province has its own local version, in accordance with local REB requirements). The online consent form does not require a signature, and completing the fields and clicking the submit button is indicative of consent. The same version of the consent form is provided to patients and caregivers; however, the patients and their caregivers each receive their own copy (sent as a PDF by email after completion online, in an encrypted, password-protected folder). The consent form is included in Supplementary Material S2. Once dyads are consented, they receive a link to complete the baseline questionnaire online.

### Allocation and randomization

Once dyads submit their baseline survey, they are randomized by the project coordinator using a computer-generated randomization schedule that uses random block sizes of 2 or 4, with an allocation ratio of 1:1, stratified by severity of HADS Anxiety subscale. SAS University Edition is used. The stratification uses the highest HADS score in the dyad categorized as “none” (HADS score < 8), “mild” (HADS score 8–10) or “moderate/severe” (HADS score ≥ 11) [42]. To ensure allocation concealment and prevent selection bias, only the study coordinator can enter the unique participant identifier [43] into the randomization schedule, along with date of baseline completion and HADS scores of both dyad members. An automated interface, programed by the study statistician, assigns the study group.

### Intervention and control groups

#### TEMPO intervention

All participants in the intervention are given access to TEMPO and continue to use all other resources offered by their cancer centre, independently of this study. The description of TEMPO follows the TiDier template [44].

**Fig. 1:**
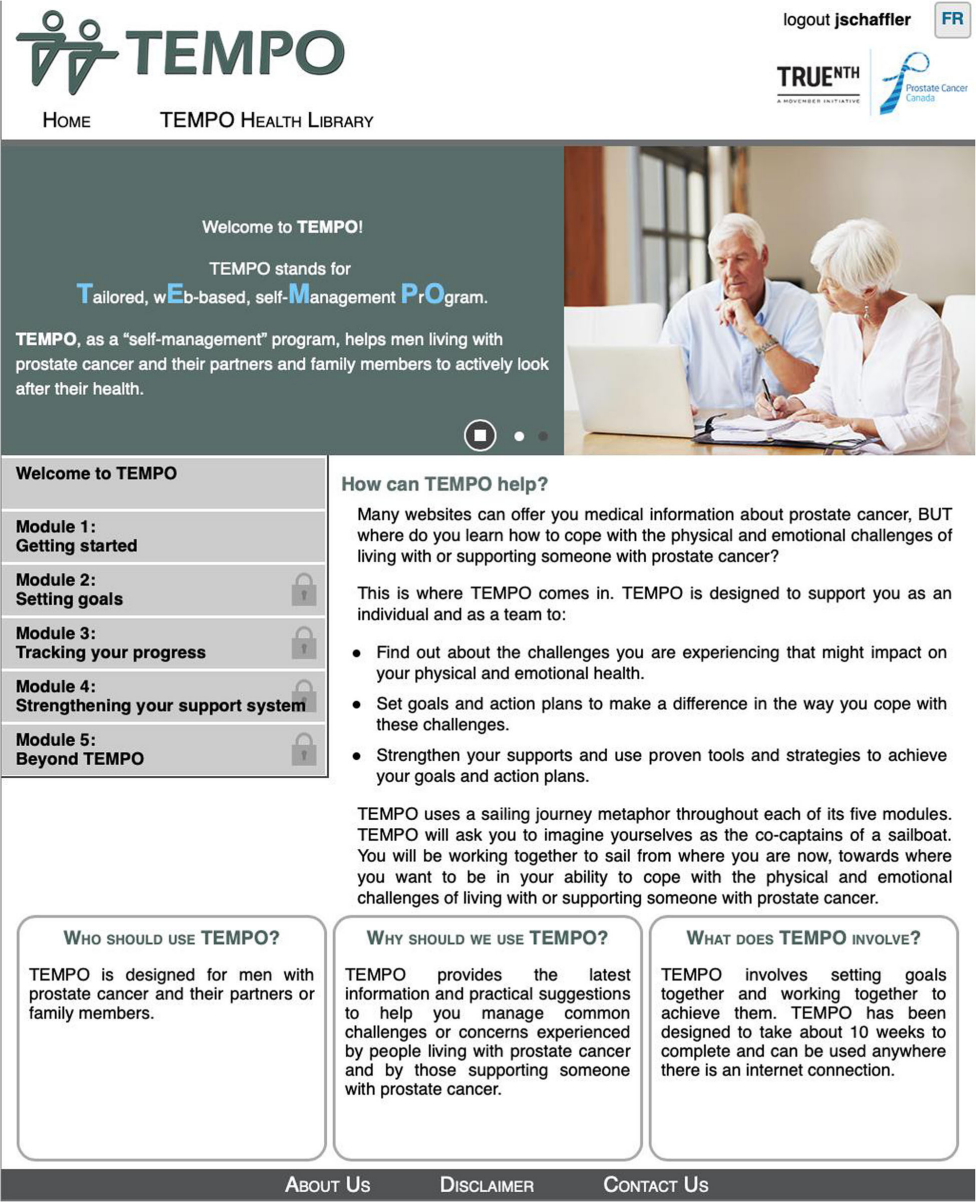
*TEMPO* landing page. This figure illustrates the content and layout of the *TEMPO* platform. Available from https://tempo.truenth.ca

**Fig. 2:**
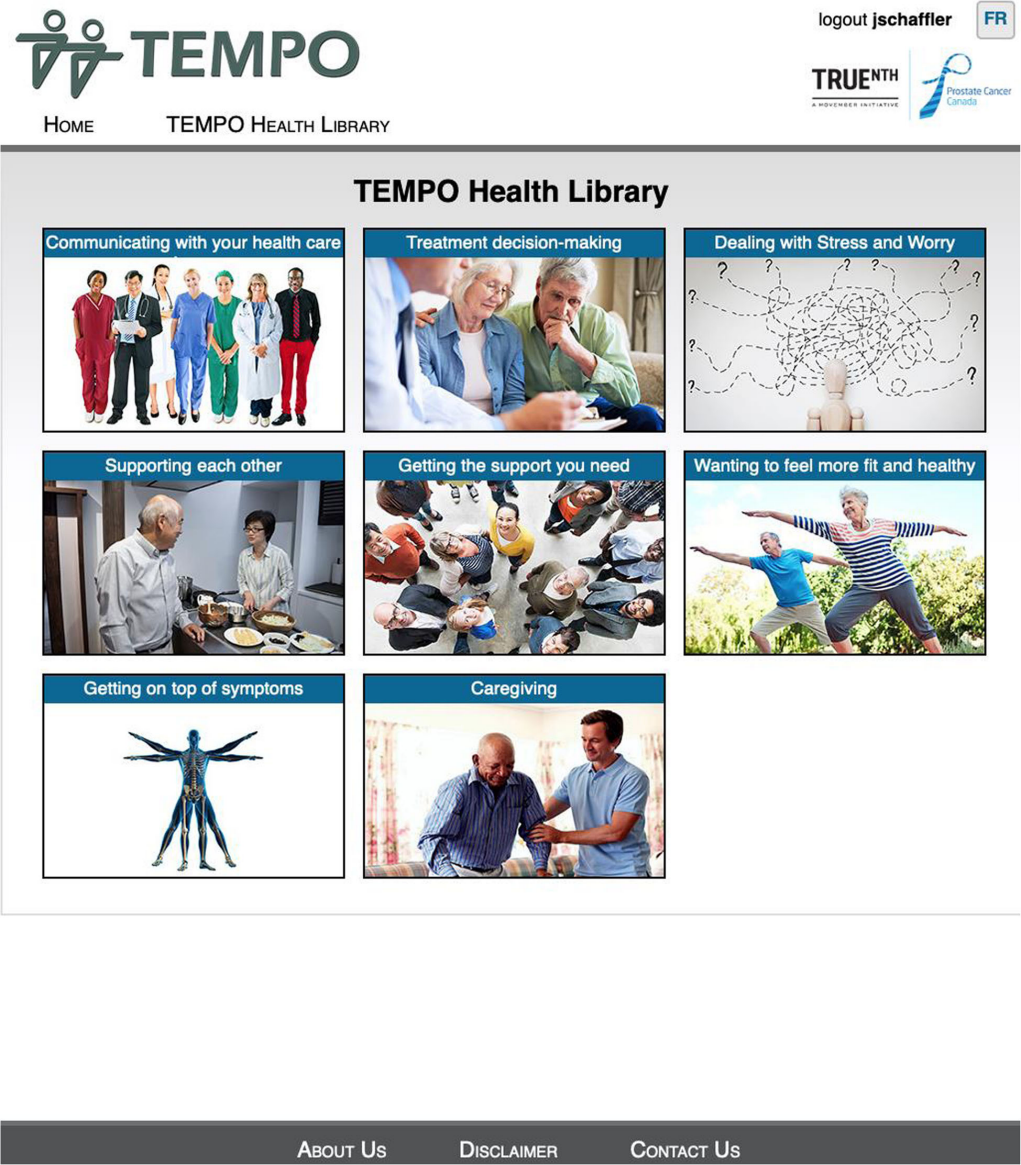
*TEMPO* Health Library landing page. This figure illustrates the layout of the *TEMPO* Health Library including the eight categories of factsheets

- *Name:* Tailored, wEb-based, psychosocial and physical activity self-Management PrOgram (*TEMPO*) [45]. *TEMPO* is a 10-week, web-based intervention (https://tempo.truenth.ca/) that complements usual care (see Fig. 1 for a screenshot of the *TEMPO* landing page).
- *Why: TEMPO* aims to increase dyads’ confidence in using self-management strategies demonstrated to be effective in addressing key psychosocial issues (e.g., dealing with stress, communicating with partner and family) and assist dyads in developing the self-regulatory skills necessary to meet the PA guidelines [46–48].
- *What:* Dyads are invited to complete five modules: (a) identification of need and priorities, (b) setting goals, (c) tracking progress, (d) strengthening your support system, and (e) maintaining behavior change beyond *TEMPO*. Modules were explicitly designed to focus on specific aspects of the behavior change process and integrate key persuasive technology techniques (e.g., primary task support) [49]. Each module specifies online (e.g., worksheets to set goals) and offline (e.g., practicing chosen self-management skills) activities. Table 1 summarizes the content and self-management skills addressed in each of the five modules.
  In addition to the modules, *TEMPO* includes a health library, incorporating 49 factsheets based on the most up-to-date evidence on self-management and PA (see Fig. 2 for a screenshot of the *TEMPO* Health Library). The content of the health library integrates content developed earlier by our team [11]. The health library includes eight sections: (a) communicating with your health care team, (b) treatment decision-making, (c) dealing with stress and worry, (d) supporting each other, (e) getting the support you need, (f) wanting to feel more fit and healthy, (g) getting on top of symptoms, and (h) caregiving. Table 2 outlines the specific topics covered in each of the sections respectively. A sample factsheet is included in Supplementary Material S3. *TEMPO* is available both in French and in English.
- *Who provides: TEMPO* is a self-directed intervention, whereby no external guidance is provided, and all the support to navigate the intervention is included in its design. TEMPO was developed by a multi-disciplinary research team, in close collaboration with community organizations, clinicians, health care managers and end users. All collaborators have experience in improving patients’ and caregivers’ adjustment to cancer.
- *How:* Participants randomized to TEMPO are sent an email with a brief, illustrated instructional guide on creating a TEMPO account (e.g., registration), and on accessing the modules (e.g., website navigation). They are also invited to schedule a phone call with a RA to review the registration instructions, receive support with account creation, and/or receive assistance with module access as required. Any comments or issues reported by participants relating to registration or access is recorded and shared with the research team.
  Although patients and caregivers might initiate the modules together, they then can progress at their own speed through the content. Once dyads identify their needs, they use the appropriate factsheets to get ideas for self-management strategies to address these and set their goals accordingly.
- *When and how much:* Participants are advised to wait approximately 2 weeks in-between each module, to pace their learning; however, based on our initial acceptability study [26], each module becomes immediately available upon completion of the preceding module. In the event that participants are completing the modules at a slower than expected pace (2 weeks per module), a reminder email is sent to check-in and offer additional support. If no response is received, and no additional module completion is observed, a second follow-up email is sent 1 week later.
- *Modifications:* Following our initial acceptability study [45], new health library content was added to help participants engage in PA during COVID-19-related confinement if outside activities are not deemed feasible. Although we had planned to send dyads pedometers and exercise bands, due to COVID-19, this was not possible. Dyads are encouraged to use their smartphone and other household equipment to support their PA.

**Table 1:**
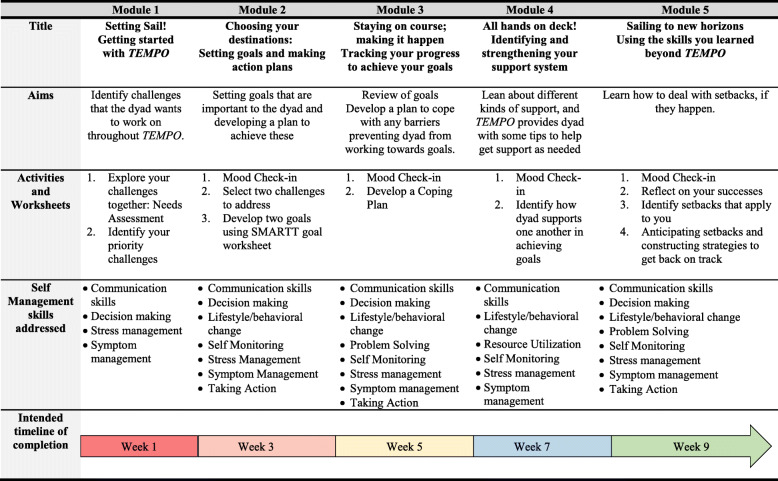
Overview of the five *TEMPO* Modules

**Table 2:** *TEMPO* Health Library Topics of the 49 Factsheets

Communicating with your health care team (***n*** = 6)	Treatment decision making (***n*** = 6)	Dealing with stress and worry (***n*** = 5)
1. Knowing what to expect after a cancer diagnosis or treatment 2. Asking questions to the health care team 3. Knowing the role of the different health professionals involved 4. Understanding what the health care professionals are telling us 5. Having our main concerns addressed during appointments 6. Telling the health care professionals what is going on	1. Making decisions about treatment 2. We do not have enough options 3. Do not understand enough 4. Telling the health care team which decisions we want 5. Feeling pressured to make decisions 6. Managing stress due to treatment-related delays	1. Feeling tense, anger, or stress 2. Feeling worried or uncertain 3. Feeling sad, down, or isolated 4. Feeling unmotivated or low on energy 5. Feeling overwhelmed
**Supporting each other** **(*n*** **= 6)**	**Getting the support you need (*n*** **= 7)**	**Wanting to feel more fit and healthy (*n*** **= 9)**
1. Helping each other feel better 2. Talking about the hard stuff 3. Dealing with conflict 4. Stressed by the changes in our roles and responsibilities 5. Feeling less connected to each other 6. We are different in how much we want to talk about the situation	1. Knowing where to go for more support^a^ 2. Getting more information about cancer and treatment 3. Asking for help from the right health professionals 4. Needing more help at home 5. Getting emotional support 6. Finding financial help 7. Finding legal help	1. Fitting physical activity into our day 2. Exercising and being physically active safely 3. Wanting to be physically active on a budget 4. Getting ideas about how to get physically active 5. Knowing how much physical activity 6. Becoming or staying motivated to do physical activity 7. Knowing how to use a pedometer 8. Knowing how to do resistance training at home 9. Aerobic exercises at home
**Getting on top of symptoms** **(*n*** **= 7)**	**Caregiving** **(*n*** **= 3)**	
1. Pain 2. Fatigue 3. Loss of appetite 4. Diarrhea 5. Incontinence 6. Erectile dysfunction 7. Symptom diary	1. Canadian Cancer Society pages on caregiving 2. Canadian Cancer Society pages on support for caregivers 3. Video testimonial from a caregiver, hosted by the Canadian Cancer Society	

^a^Sample factsheet of “Knowing where to go for more support” is included in Supplementary Material S3. Each factsheet uses a similar format and is similar in length

#### Control group

A wait-list control group is used, whereby dyads do not receive access to *TEMPO* initially, but have access to all the resources available at and referred to by their respective participating cancer treatment centers. These dyads have access to support and information provided by multi-disciplinary teams at their treatment centers. Depending on the center, they may also be referred to supportive resources provided by the Canadian Cancer Society or by TrueNTH Canada (an initiative of Prostate Cancer Canada). In addition, they may be encouraged to use local rehabilitation and support group resources for patients and caregivers. A 3-month follow-up questionnaire captures information on the types of support resources that the dyads had been referred to and used. Once these dyads have completed the follow-up questionnaire, they are sent the login information for *TEMPO.*

### Theoretical frameworks that guided the development of *TEMPO*

- *Stress and Coping Framework* [50] assumes that individuals who cope better with cancer challenges experience less anxiety. Active coping generally leads to positive adjustment [51], whereas avoidant coping is linked to higher anxiety [52].
- *Framework of Dyadic Coping* [53] recognizes the impact of the patient–caregiver relationship on each person’s anxiety, whereby positive dyadic coping (e.g., supportive communication) enhances patients’ and caregivers’ reported outcomes [54].
- *Self-efficacy theory* [55] posits that people are more likely to engage in activities they feel confident doing, with self-efficacy-enhancing strategies including achievement of behavioral goals, behavior modeling by similar others, and verbal persuasion.

### Blinding

Participants are not blinded to group allocation, as the conditions are described in the consent form. However, they are blinded to specific pilot outcomes, reducing potential response biases [56]. Investigators are blinded to group allocation until the database is locked. The coordinator randomizes dyads and cannot be blinded to group allocation. The RAs recruiting participants do not have access to the randomization schedule or to randomized participants.

### Data collection

Participants complete an eligibility screening checklist (T0) and two questionnaires: baseline (T1) and 3 months later (T2). Upon return of their T2 questionnaire, participants are then invited to participate in a dyadic, semi-structured exit interview to further assess acceptability. Data are also collected through the local recruitment logs (covering all steps up to consent), the overall study log (follow-up of enrolled and randomized dyads), and website tracker information.

#### Eligibility screening interview (T0)

A checklist was developed to ensure patients are eligible. Patients must confirm that they are interested in the study, have received (within the last 24 months) or are planning to receive the eligible prostate cancer treatments previously described, that they have a caregiver, and that they have access to the internet. Caregivers must confirm that they are interested and that they have not been diagnosed with cancer themselves in the last year. Reasons for refusal or ineligibility are recorded in the local recruitment logs.

#### Baseline (T1) questionnaire

The online baseline (T1) questionnaire is available in French and English and is completed online via SimpleSurvey by patients and caregivers (separately). The baseline questionnaire includes the following self-administered questionnaires to capture the primary and secondary outcomes of interest. Table 3 includes a summary of the Cronbach’s alpha for each scale.

**Table 3:** Cronbach’s alpha’s for measures to be used

Measures	Cronbach’s alpha^a^ measure of reliability
**Physical and emotional outcome measures**	
Medical Outcome Survey (MOS) Short-Form (SF-12) [57]	
Physical Component Summary (PCS)	α = 0.82 [60]
Mental Component Summary (MCS)	α = 0.75 [60]
Hospital Anxiety and Depression Scale (HADS) [42]	α = 0.68–0.93 [62]
Perceived Stress Scale (PSS) [68]	α = 0.75–0.89 [69]
**Self-management outcome measures**	
Health Education Impact Questionnaire (heiQ) [71]	
Constructive attitude and approaches	α = 0.81 [71]
Skill and technique acquisition	α = 0.81 [71]
Health Service navigation	α = 0.82 [71]
Positive and active engagement in life	α = 0.86 [71]
Health-directed activities	α = 0.80 [71]
Self-monitoring and insight	α = 0.70 [71]
Health-directed behaviors (caregivers only)	α = 0.80 [71]
Emotional well-being (caregivers only)	α = 0.89 [71]
Health Literacy Questionnaire (HLQ)	
Having sufficient information to manage my health	α = 0.89–0.94
Actively managing my health	α = 0.76–0.88
**PA & exercise outcome measures**	
International Physical Activity Questionnaire [76]	ρ =0.80 [76]
Physical Activity Plan and Intention	---
Multidimensional Self-Efficacy for Exercise Scale (MSES) [78]	
Across dimensions of task, coping, and scheduling	α = 0.83–0.91 [78]
Across time (baseline, 6-weeks, and 12-weeks)	α = > 0.78 [78]
**Dyadic coping outcome measures**	
Dyadic Coping Inventory (DCI) [79]	
Across both patients and partners	α = 0.63–0.84 [80]
°Revised Dyadic Adjustment Scale (RDAS) [82]	α = 0.89–0.95 [83]

^a^ Cronbach’s alpha is interpreted as follows: Excellent (α ≥0.90), Good (α = ≥0.80-0.89), Acceptable (α = ≥0.70-0.79), and Poor (α = <0.70).

° Optional scale for both patients and caregivers to complete

#### Medical Outcomes Study (MOS) 12-item Short Form Health Survey (SF-12) [57]

The SF-12 yields a Physical Component Summary (PCS) and a Mental Component Summary (MCS) score [57]. This scale has been used in other caregiver studies [5, 58, 59]. The scores are calculated using weighted scoring and standardized from 0–100 (US norm mean = 50, SD = 10). Internal consistency of the SF-12 PCS (α = 0.82) and MCS (α = 0.75) have been assessed to be adequate [60] across nine European countries including France [61].

#### Hospital Anxiety and Depression Scale (HADS) [42]

The 14 items on the HADS are equally divided between the HADS-Anxiety and HADS-Depression subscales (α = 0.68–0.93) [62]. Subscale scores of 8 to 10 are categorized as borderline and scores of 11 to 21 as clinical [42]. A recent review identified the HADS as one of the questionnaires of choice to assess anxiety and depression among patients with cancer [63]. A criticism of the HADS, mainly based on classical test theory analyses, is that it is not a dependable means of differentiating anxiety and depression [64]. However, studies by our team using item response theory [65, 66] have supported the ability of the HADS to discriminate between anxiety and depression among patients with cancer and their caregivers. Furthermore, the reliability and discriminant validity of the French Canadian version of the HADS remains high (α = 0.79–0.89) [67].

#### Perceived Stress Scale (PSS) [68]

The PSS consists of 10 items that measure the degree to which participants appraise situations in their life as stressful. Participants are asked about the frequency of their feelings and thoughts during the last month. Each question is rated on a 5-point Likert scale (0 = never, 1 = almost never, 2 = sometimes, 3 = fairly often, 4 = very often). Scores are calculated by reversing the scores (e.g., 0 = 4, 1 = 3, 2 = 2) on the four positive items. A summary score is then calculated by summing all 10 items. The PSS-10 has adequate reliability and validity across numerous studies (α = 0.74–0.91) [69] and in the French language (α = 0.73–0.81) [70].

#### Health Education Impact Questionnaire (heiQ) [71]

The heiQ (Version 3.0) is a 40-item measurement system examining the effects of health education programs on individuals’ self-management skills for coping with a chronic condition [71]. It consists of eight different subscales: (a) positive and active engagement in life, (b) skill and technique acquisition, (c) constructive attitude and approaches, (d) self-monitoring and insight, (e) health services navigation, (f) social integration and support, (g) health-directed activity, and (h) emotional distress [71]. Given the aim of *TEMPO*, patients are given six subscales (subscales: a, b, c, d, e, f), whereas caregivers complete the entire scale. Studies have confirmed its reliability and validity across subscales (α = ≥ 0.70), in the French language (α = 0.69–0.89) [71, 72] and in the cancer setting [73] (α = 0.75–0.90).

#### Health Literacy Questionnaire (HLQ )[74]

The 44-item HLQ assesses health literacy across nine conceptually distinct domains. Patients and caregivers both complete the domains most relevant to *TEMPO*: having sufficient information to manage my health (4 items), and actively managing my health (5 items). The HLQ has been found to possess adequate validity and reliability across these subscales (α = 0.76–0.94) [74] and in the French language (α = 0.77–0.91) [75].

#### International Physical Activity Questionnaire-Short Form (IPAQ-SF) [76]

The IPAQ-SF is a 7-item self-reported measure to assess the frequency and duration of vigorous-/moderate-intensity PA, walking, and sitting respectively. Activities must be performed for at least a duration of 10 min per session, and within the past 7 days to be reported. It has been found to be valid and reliable across 12 countries, with an overall Spearman’s ρ of 0.80 [76].

#### Physical Activity Plan and Intention [77]

The PA plan and intention questionnaire consists of 8 items developed by our team based on recommendations from Ajzen [77]. It aims to capture indicators of (a) participants’ planned PA behaviors (e.g., where, when, what, and how) and (b) participants’ intentions towards increasing their PA and meeting the recommended physical activity guidelines. Participants rate their level of agreement with 4 statements pertaining to planning their PA (e.g., “I have made a detailed plan about where I will engage in physical activity over the next week.”) and 4 statements pertaining to their intentions to carry out their planned behaviors (e.g., “I intend to work towards the physical activity guidelines to meet them in the next four weeks.”). Rating is done on a 7-point Likert Scale (1 = strongly disagree to 7 = strongly agree).

#### Multidimensional Self-Efficacy for Exercise Scale (MSES) [78]

The 9-item MSES measures participants’ self-efficacy for exercise participation. Three questions (all of which begin with the root phrase “How confident are you that you can…”) assess particular aspects of exercise behavior across three dimensions respectively: (a) task (e.g., follow directions to complete exercise), (b) coping (e.g., exercise when you lack energy), and (c) scheduling (e.g., include exercise in your daily routine). Participants rate their level of confidence to complete each item on a 100% confidence scale, ranging from 0 = not confident at all to 100 = completely confident. Studies [78] evaluating the MSES have assessed its reliability and validity to be good across each respective dimension (α = 0.83–0.91) and across time (α = > 0.78 at baseline, 6 weeks and 12 weeks).

#### Dyadic Coping Inventory (DCI) [79]

The 37-item DCI (patients and partners; α = 0.63–0.84) [80] is a self-reported measure that captures how partners support one another in response to individual and collective stressors [81]. Items are rated on a 5-point Likert scale (1 = very rarely, 2 = rarely, 3 = sometimes, 4 = often, and 5 = very often). The eight negative items are reverse coded, and subscale and summary scores are calculated. The DCI has been found to be reliable and valid for use across 25 different languages [81].

#### Revised Dyadic Adjustment Scale (RDAS) [82]

This is a 14-item questionnaire capturing dyadic consensus, satisfaction, cohesion, and affective expression. Cronbach’s alpha across the subscales range from 0.89 to 0.95 [83]. The DAS-revised is a widely used measure of satisfaction with intimate relationships. Completion is optional for both patients and caregivers.

#### Use of Healthcare services and change in employment

This 9-item questionnaire previously developed by the team [84] captures use of (a) consultation with health care professionals, (b) hospital admissions, (c) medications purchased, (d) use of community services, (e) costs related to medical care, (f) change in employment, (g) change in number of hours worked, and (h) performance at work.

#### Follow-up (T2) questionnaire

All patients and their caregivers complete the follow-up questionnaire separately. Along with the same measures included in T1, the T2 questionnaire includes (a) a health services and community-based resources survey to monitor usual care and co-interventions, (b) a *TEMPO* use and acceptability questionnaire based on previous acceptability measures [27, 40, 85], and (c) The System Usability Scale (SUS) [86]. The answers to each item of the *TEMPO* use and acceptability questionnaire will be examined individually and mostly used to guide the exit interviews. The SUS consists of 10 items assessing the usability of a website. Participants rate aspects of the website design on a 5-point Likert scale (1 = strongly disagree to 5 = strongly agree).

#### Dyadic exit interviews

Dyads where at minimum one member returned their T2 questionnaire are invited to participate in a semi-structured, telephone or online (using Microsoft Teams) exit interview. Interview questions focus on overall experience and usefulness, goals set, progress towards achieving goals, time spent on *TEMPO*, and self-management skills learned through *TEMPO*. An RA with training and experience in qualitative methodology as well as familiarity with *TEMPO* conducts the interviews. The interviews are anticipated to last 40–60 min and are audio-recorded and transcribed verbatim.

#### Study log

The following information is collected by the local RAs: (a) number of individuals approached, (b) number of individuals self-referred to the study, (c) number of individuals eligible and ineligible, (d) number of individuals who declined participation (with reason). The overall study log managed by the study coordinator includes (e) number of participants consented and randomized, (f) number of participants who withdrew (with reason), and (g) number of participants who dropped out (with reason).

#### User tracker information

User tracking monitors adherence to *TEMPO*. These data include: number of logins, time spent on each module, number of times logged in each module, modules completed (clicked through the module), and worksheets completed.

### Data analysis

SAS University Edition, STATA 15 and R version 3.1.2 software will be used for data analysis.

#### Feasibility data

Recruitment and refusal rates along with their respective 95% confidence interval will be calculated. The proportion of missing data will also be similarly calculated.

#### Acceptability data

Average score on the SUS will be calculated, where a score of 68 will be considered high [86]. The proportion of patients who withdrew and the 95% confidence interval will be calculated. Adherence will be calculated based on the number of modules patients and caregivers completed. A module will be considered complete if the user clicked through the entire module and spent at least 15 min on it. Adherence will be categorized as non-adherent (1–2 modules completed), moderate adherence (3–4 modules completed), or high adherence (5 modules completed).

#### Clinical significance

An intention-to-treat analysis will be conducted and missing data will be accounted for via multiple imputation. Baseline imbalances across intervention and control groups will be examined for each outcome score. As this is a pilot trial, effect sizes to estimate the clinical significance of the interventions will be calculated by computing differences between two estimated means divided by the pooled standard deviation [32]. In addition, the proportion of participants that improved on the primary outcome measures by at least the minimal clinically important difference (MCID) will be calculated. The MCID for patients with cancer and their caregivers for the HADS and SF-12 are not available; however, validated MCIDs in other populations will be used. For the HADS, the MCID that will be used is 1.5 [87] and for the SF-12 MCS 3.8 [88] and PCS 3.3 [88]. Subsequent inferential statistics will be carried out for exploratory purposes only to inform the planning of the larger trial. In the event that groups are imbalanced at baseline, they will be compared using regression analysis where the baseline scores will be included in the model as a covariate. No corrections will be undertaken for multiple testing due to the exploratory nature of this pilot.

#### Health economic analysis

This analysis will take the form of a cost–utility analysis to compare the difference in total costs and health utility weighted outcomes between competing alternatives. Costs will be calculated from a societal perspective, and will include the costs of program delivery, costs of health system resource utilization, and patient–caregiver indirect costs. Health utility represents the preference that an individual places on a given state of health. Responses to the MCS and PCS scores of the SF-12 will be converted to utilities [89]. Utilities can be converted into quality-adjusted life years (QALYs) by multiplying the health state utility by the amount of time a person spends in that state. Expressing outcomes in terms of QALYs allows for the comparison of incremental effectiveness between competing alternatives where survival is not expected to differ, but there is an expected difference in QOL. Total cost and QALYs from each group will be compared to calculate the incremental costs and effectiveness of the intervention relative to usual care, which will be used to calculate incremental cost-effectiveness ratios. Decision uncertainty analysis will be conducted using non-parametric bootstrapping techniques, and cost and effectiveness differences will be used to produce cost-effectiveness acceptability curves. Statistical uncertainty will be plotted on the cost-effectiveness plane.

#### Analysis of exit interview data

Exit interview transcripts will be analyzed in QSR NVivo using thematic analysis [90]. Initially, words or statements related to feasibility and acceptability will be extracted from the transcripts by assigning a code. Comparison of codes across transcripts will identify similarities and differences, which will lead to the identification of themes. Transcripts will be coded independently by two RAs to enhance credibility [91].

### Adverse event and protocol deviation reporting

Any serious adverse event occurring to a research participant will be reported to the lead research ethics committee without delay. Any protocol deviations will be recorded and depending on the nature, will be submitted to the ethics committee as an amendment.

## Discussion

Caregivers of men with prostate cancer remain a vulnerable group throughout the illness trajectory, as they often experience more anxiety and needs than patients [3, 6], but have access to fewer services [11]. This means that they are at high risk for both physical and emotional problems [1–3, 5, 6]. Despite some advocacy efforts, cancer care resources are already too stretched to respond to caregivers’ growing support needs. In support, a recent review of the costs of informal cancer care provision estimates that caregivers provide nearly $5,000 worth of care per month [4]. Although this significantly alleviates the demands on the healthcare system [4], it leaves caregivers particularly vulnerable to poor health outcomes. Thus, it is evident that there is a critical demand for studies testing the effectiveness and sustainability of interventions aimed at preserving and/or improving caregivers’ QOL. Pilot findings will not only be published, but will also be communicated to participants as a newsletter and/or a short video.

Although the number of web-based, self-management interventions is increasing [92], many fail to incorporate a dyadic approach to treatment and/or lack self-management strategies such as PA to target caregivers’ physical health [13, 14, 25]. Thus, *TEMPO* attempts to address these shortcomings. The present study primarily aims to test the feasibility and acceptability of *TEMPO* among men with prostate cancer and their caregivers. We posit that by targeting multiple risk factors of caregiver burden [5, 6], and combining the best evidence in terms of coping skills, PA training, and self-management, *TEMPO* has a great potential impact on clinical outcomes. In addition, the design and evaluation of this pilot trial was informed by the Medical Research Council’s (MRC) framework for complex interventions [93, 94], and is based on extensive prior development work, including the engagement of key stakeholders [26].

The proposed study protocol has some limitations. Notably, recruitment and participation is occurring during the novel COVID-19 pandemic, lending to specific challenges including the following: (a) recruitment may be delayed due to lack of identification of eligible dyads by hospitals, (b) eligible dyads may be those who are healthier and/or perceive themselves at lower risk of contracting the novel virus (and thus are more willing to participate), (c) participants may be limited in the extent/variety of their PA activities due to confinement measures, and (d) measures such as the HADS-Anxiety, HADS-Depression, PSS, and those pertaining to exercise intensity and self-efficacy may be artificially inflated or decreased as a result of the pandemic restrictions. Additional limitations include potential selection biases identified during the initial *TEMPO* acceptability study [26], namely participating dyads tended to be who are retired, and capable of engaging in medium to high intensity PA, and those who reported supportive dyadic relationships. A number of mitigation strategies have been considered and are being employed to address these challenges. The research team has developed, in consultation with the ethics committee, a comprehensive online recruitment strategy, including an online consent form. Additional suggestions for home-based activities and exercises have been added to the *TEMPO* health library, to reduce emphasis on activities undertaken outside of the home. The RAs who provide interface support to *TEMPO* participants have been informed of additional community-based resources that the dyads might benefit from during the pandemic. The semi-structured exit interviews contain questions that intend to tap into the pandemic-related limitations experienced by dyads to contextualize quantitative results.

In conclusion, our feasibility and acceptability analyses will begin to develop the knowledge base on *TEMPO*’s value for men with prostate cancer and their caregivers. Furthermore, the results will directly contribute to the design of a larger trial. Additionally, it lays the foundation on which future research can examine the impacts of web-based psychosocial and PA self-management programs among other dyadic populations coping with illnesses along a care continuum.

## Supplementary Information

**Additional file 1.** CONSORT and SPIRIT Checklists.

**Additional file 2.** Consent forms.

**Additional file 3.** Sample factsheet.

## Data Availability

Most of the data generated or analyzed during this study are included in this published article (and its supplementary information files). For any additional datasets used and/or analyzed during the current study, they are available from the corresponding author on reasonable request.

## Abbreviations

DCI: Dyadic Coping Inventory
ES: Effect size
HADS: Hospital Anxiety and Depression Scale
heiQ: Health Education Impact Questionnaire
HCP: Healthcare provider
HLQ: Health Literacy Questionnaire
IPAQ-SF: International Physical Activity Questionnaire-Short Form
MCS: Mental Component Summary (of the MOS SF-12)
MOS SF-12: Medical Outcome Survey Short-Form-12
MRC: Medical Research Council
MSES: Multidimensional Self-Efficacy for Exercise Scale
MUHC: McGill University Health Centre
PA: Physical activity
PCC: Prostate Cancer Canada
PCS: Physical Component Summary (of the MOS SF-12)
PSS: Perceived Stress Scale
PID: Participant identifier
QALYs: Quality-adjusted life years
QOL: Quality of life
RA: Research assistant
RDAS: Revised Dyadic Adjustment Scale
REB: Review Ethics Board
SHSC: Sunnybrook Health Sciences Centre
SUS: System Usability Scale
TBCC: Tom Baker Cancer Centre
*TEMPO*: Tailored, wEb-based, psychosocial and PA self-Management PrOgram
US: United States
VGH: Vancouver General Hospital
