# Supplementary material for: A study protocol for a multicenter randomized pilot trial of a dyadic, tailored, web-based, psychosocial, and physical activity self-management program (TEMPO) for men with prostate cancer and their caregivers
Source: Pilot Feasibility Stud. 2021 Mar 20;7:78. doi: 10.1186/s40814-021-00791-6 (PMC7980105; doi:10.1186/s40814-021-00791-6)
Supplement: Supplementary file 2 — Additional file 2. Consent forms. [file 40814_2021_791_MOESM2_ESM.docx]

| **Study Title** | **Evaluating *TEMPO* - a Tailored, wEb-based, psychosocial and physical activity self-Management PrOgram** | |
| --- | --- | --- |
| **Principal Investigator:**  **Co-Principal Investigator**: | **Sylvie Lambert, R.N., Ph.D.,** Associate Professor, Ingram School of Nursing, McGill University; Research Associate, St. Mary’s Research Centre  3506 University Street, Wilson Hall room 400, Ingram School of Nursing, McGill University, Montreal, Quebec H3A 2A7  **Lindsay Rosamond Duncan, Ph.D**., Assistant Professor, McGill University, Department of Kinesiology and Physical Education  McGill Sports Complex, 475 Pine Avenue West, Montreal, Quebec, H2W 1S4 | |
| **Co-Investigators:** | **Lauren Walker, Ph.D., Professor, Department of Oncology, Division of Psychosocial Oncology, University of Calgary; Clinical Psychologist, Tom Baker Cancer Centre**  **Nicole Culos-Reed, Ph.D**., Associate Professor, Health and Exercise Psychology, Faculty of Kinesiology, University of Calgary & Adjunct Professor, Department of Oncology, Faculty of Medicine, University of Calgary; Research Associate, Health and Exercise, Psychosocial Resources, Tom Baker Cancer Centre, University of Calgary  **Carmen G. Loiselle, R.N., Ph.D.,** Joint Associate Professor, Ingram School of Nursing and Department of Oncology, Faculty of Medicine, McGill; & Co-Director, Segal Cancer Centre Jewish General Hospital; Scientific Director, Hope & Cope, Jewish General Hospital  **Daniel Santa Mina, CEP, Ph.D.,** Assistant Professor, Faculty of Kinesiology and Physical Education, University of Toronto; Clinician-Scientist, Princess Margaret Cancer Centre, & Co-Chair Academic Advisory Committee  **Paramita Saha-Chaudhuri, M.D., Ph.D.,** Assistant Professor, Department of Epidemiology, Biostatistics and Occupational Health, McGill University  **Stuart Peacock, DPhil,** Associate Professor, School of Population and Public Health, University of British Columbia; Co-Director, Canadian Centre for Applied Research in Cancer Control; Scientist, British Columbia Cancer Agency  **Andrew Matthew, Ph.D.,** Assistant Professor, Faculty of Medicine, Departments of Surgery and Psychiatry, University of Toronto; Senior Staff Psychologist, Department of Surgery, Princess Margaret Cancer Centre  **Larry Goldenberg, M.D.,** Professor, Department of Urologic Sciences, University of British Columbia; Director of Development and Supportive Care, Vancouver Prostate Centre  **Janet Ellis, M.D.,** Lecturer, Department of Psychiatry, University of Toronto; Psychiatrist and Director, Psychosocial Care in Trauma, Sunnybrook Health Sciences Centre  **Anne Katz, R.N., Ph.D**., Adjunct Professor, College of Nursing, University of Manitoba; Clinical Nurse Specialist, Prostate Centre, CancerCare Manitoba  **Wassim Kassouf, M.D.**, Professor, Faculty of Medicine, McGill University; Attending Surgeon, Department of Medicine, Urology, McGill University Health Centre | |
| **Sites:** | **Montreal, Quebec:**  Montreal West Island Integrated University Health and Social Services Centre  McGill University Health Centre  **Toronto, Ontario:**  Sunnybrook Health Sciences Centre | **Calgary, Alberta:**  Tom Baker Cancer Centre  **Vancouver, British Columbia**  Vancouver Prostate Centre |
| **Funding & Amount:** | Prostate Cancer Canada  Amount: $ 434, 040 | |
| **Protocol number:** | MP-CUSM-15-179 | |
| **Conflict of interest:** | None | |

**Introduction**

Men with prostate cancer and their caregivers are invited to participate in a research study that will evaluate a web-based, coping skills training and in-home exercise program. This new program is called *TEMPO* - a Tailored, wEb-based, psychosocial and physical activity self-Management PrOgram.

The present document contains detailed information about this research study. Its purpose is to explain to you as openly and clearly as possible all of the aspects of this study. Before you accept to participate in the study, please take the time to carefully read all the information below. This form may contain some words or ideas that you do not understand. If you have any questions, we invite you to ask the research assistant or investigators responsible for this study to explain anything that you find unclear. You may discuss the study with a person you trust before making your decision. If you decide to participate, you will be asked to check the relevant box and provide your name and contact information on the last page of this document (declaration of consent). A copy of this information sheet and the finalized consent form will be emailed to you for your records.

**What is this research about?**

The research team has developed a web-based coping skills training and in-home physical activity program designed to meet the needs of men with prostate cancer and their caregivers. The team worked with Prostate Cancer Canada to develop this program and will ask 80 men with prostate cancer and their caregivers to complete the website modules and related activities as much as possible over 7-10 weeks, and complete a questionnaire at the time of registration, and 3 months after registration. You may also be asked to complete one exit interview. This information will help us determine the suitability and value of this program, including what kind of changes we need to make to ensure that it can be successfully used by men with prostate cancer and their caregivers.


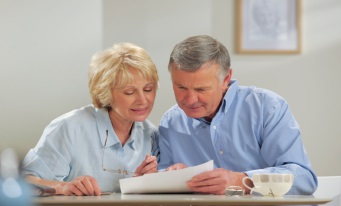


**Who can participate in this study?**

We are recruiting 80 men with prostate cancer and their caregivers. As much as possible, we are hoping patients and caregivers will participate in this study together, but this is not a requirement.

**Men with prostate cancer are eligible if they:**

- Plan to, are undergoing currently, or who have undergone (within the last 24 months) active treatment (i.e., surgery, chemotherapy, radiotherapy, and/or hormonotherapy). Unfortunately, men under active surveillance or watchful waiting are not eligible; and
- Have an eligible primary caregiver who is willing to participate in the study. In the context of this study, a caregiver is defined as a family member, spouse, adult child, friend, or any other significant person involved in providing unpaid assistance (e.g., emotional support, practical assistance) to someone with prostate cancer; and

**Caregivers are eligible if they have:**

- Been identified by the patient as his primary source of support;
- Not been diagnosed and have not undergone treatment for cancer in the previous year; and

Both patients and caregivers must have access to the internet, be fluent in English or French, and be able to participate in some physical activity, within their limits and in accordance with any guidelines given by their respective physicians.

Participants will be recruited from across Canada, including from the, McGill University Health Centre (MUHC), , Tom Baker Cancer Centre (TBCC), Vancouver General Hospital (VGH), , and Sunnybrook Health Sciences Centre (SHSC) sites.

**What are you being asked to do?**

This study has two parts. Consenting patients and their caregivers will first be assigned to one of two groups by a process called randomization, which means that there is a 50% change that you will be in either Group A or Group B.

**Group A**: You will continue to receive all the care that you are entitled to at your treating centre, including medical and supportive cancer care. However, in addition, you will be asked to complete a questionnaire at the same time as signing this consent form. You will also receive access to *TEMPO*, the online intervention and complete its modules and the related activities over 7 to 10 weeks. At the end of this period (3 months after completing the registration questionnaire) you will be asked to complete a second questionnaire. We will also ask you to partake in a brief interview to get your feedback on *TEMPO.*

**Group B**: You will continue to receive all the care you are entitled to at your treating centre, including medical and supportive cancer care. In addition, you may use any resources available to you at your treating centre. You will be asked to complete a questionnaire at the same as signing this consent form. You will then be contacted three months later to complete a second questionnaire. Upon completing this second questionnaire, you will get access to the TEMPO website as a thank you for participating. You are welcome to give us feedback on the website after this but no additional formal questionnaires will be required.

**How much time will it take?**

- Entire duration of study participation:
- If you are in Group A, the website will take approximately 7 to 10 weeks to complete
  - You will fill out a questionnaire at the same time as the consent form (approximately 30-45 minutes) and then be contacted three months after you complete the study to fill out a questionnaire (approximately 30-45 minutes))
  - You will be invited to participate in one, optional exit interview (approximately 60-90 minutes).
- If you are in Group B, you will fill out a questionnaire at the same time as the consent form (approximately 30-45 minutes) and then be contacted three months after you complete the study to fill out a questionnaire (approximately 30-25 minutes each time or 60-90 minutes total).
- Using the information resources: Time will vary for each patient and caregiver

**What are the benefits of participating?**

You may benefit from participating in this research study, but we cannot guarantee it. *TEMPO* was designed to provide the most up-to-date information to enhance patients’ and caregivers’ ability to manage the challenges they face and improve upon their health and functioning. Your feedback will help the research team to improve *TEMPO* and ensure it is designed in a way that will best support patients and their caregivers through the challenges of cancer. Also, the information you provide will be used to develop recommendations that may guide cancer organisations as they refine their support services in the future.

**What are the risks of participating?**

This study does not involve any drugs, blood tests, or physical examination. Therefore, it is expected that participants will face minimal risks during this study. One potential risk is that your muscles might feel sore after participating in physical activity. Another potential risk is that you may feel discomfort in openly expressing your opinions to strangers. We only expect you to say what you feel comfortable sharing. You may withdraw from the study at any time. If you have any concerns about participating, we advise you to contact the research team or your doctor. The Canadian Cancer Society also has a helpline that can provide you with any additional support you may need (1-888-939-3333).

**What choice do you have?**

Your participation in this study is voluntary. Therefore, you may refuse to participate. You may also withdraw from the ongoing project at any time, without giving any reason, by informing either the Research Assistant or the principal investigators (S. Lambert or L. Duncan). Your decision not to participate in the study, or to withdraw from it, will have no impact on the quality of care and services to which you are otherwise entitled. You will be informed in a timely manner if any information becomes available that may impact your willingness to continue participating in this study. If you withdraw or are withdrawn from the study, the information already collected about you during the study will be destroyed if it can be identified as yours. If the data has been anonymized or was always anonymous (e.g. does not contain any information that can be used to identify you), the data will continue to be used in the analysis of the study.

**How will your privacy be protected?**

During your participation in this study, the research team will collect and record information about you in a study file. We will only collect the information required to meet the study’s scientific goals. All the information collected during this study will remain confidential to the extent provided by law. For auditing purposes your study file may be examined by individuals mandated by the funder, the McGill University Health Centre, or the Research Ethics Board. All these individuals adhere to policies on confidentiality. To protect your identity and the confidentiality of your personal information, you will only be identified by a code number. The key to the code linking your name to your study file will be kept by the researcher in charge of this study. All study data will be kept for seven years and the destroyed.

All audio-recordings will be transcribed (your words will be written down) in a de-identified fashion (i.e. your name will not appear in the transcripts). The audio-recordings will then be destroyed. It is possible that direct quotes of what you said will be presented in publications and/or conferences. However, precautions will be taken to ensure that it will not be possible to identify you.

According to the Quebec *Act respecting Access to Documents held by Public Bodies and the Protection of Personal Information, R.S.Q., chapter A-2.1*, you have the right to consult your study file to verify the information or to have it corrected, if necessary. You may use this right as long as the principal investigator or the institution holds this information. However, to protect the scientific integrity of the research study, there may be certain information that you can only access after this study has ended.

**Who is funding th** **is research study?**


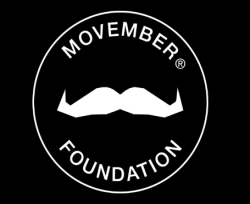
**
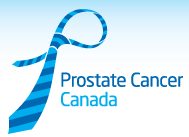
**

TEMPO was awarded by Prostate Cancer Canada and is proudly funded by the Movember Foundation.

**Is there compensation for participating?**

Your participation in this study should not result in any extra costs to you. As a token of appreciation, you will receive $30.00 for your participation in this study.

**Is there compensation in case of injury?**

If you should suffer any injury following any procedure or activity related to the research project, you will receive the appropriate care and services for your medical condition, as covered by your provincial healthcare plan, without any charge to you. By accepting to participate in this research study, you are not giving up any of your legal rights nor removing any civil or professional responsibility from the researchers or the institutions.

**How will the information collected be used?**

The information collected will primarily be used to determine the value of *TEMPO*. The results of this study may be presented at scientific meetings or published in medical journals, but your identity or any other identifying information will not be revealed in any publication or report. The data may also be used to promote *TEMPO* and for further analysis related to the study or to help in the development of future research studies.

If you would like a summary of the results mailed to you at the end of the study, please indicate this on your consent form.

**If I want more information, who should I contact?**

If you have questions about the study please contact: principal investigator, Dr. Sylvie Lambert, by e-mail at sylvie.lambert@mcgill.ca or the project coordinator, Manon de Raad, at manon.deraad@ssss.gouv.qc.ca

If you have a problem or question about your rights while taking part in this study or if you have comments or want to file a complaint, please contact the Hospital Complaint Commissioner/ Ombudsman:

**McGill University Health Centre:**

Montreal General Hospital: 514 934 1934, ext. 44285

Glen/Royal Victoria Hospital: 514 934 1934, ext. 35655

Mail: MUHC Office of the Ombudsman, 1650 Cedar Room E6.164, Montreal, Qc H3G 1A4.

E-mail: [ombudsman@muhc.mcgill.ca](mailto:ombudsman@muhc.mcgill.ca)

**Oversight of the ethical aspects of the research study**

The MUHC Research Ethics Committee (REC) of the participating hospitals approved this research study and is responsible for the ethics oversight of this project for all Quebec RSSS Institutions. Any change or amendment made to the research protocol or to the information and consent form must first be approved the MUHC Research Ethics Committee.

| **Evaluating *TEMPO* - a Tailored, wEb-based, psychosocial and physical activity self-Management PrOgram** |
| --- |

**I. Participant’s statement of consent**

I have reviewed the information and consent form. I acknowledge that the research study was explained to me, that I am satisfied that my questions were answered, and that I was given enough time to make a decision.

I agree to participate in this research study according to the conditions stated above, including having my interviews audio-recorded. I authorize the research team to collect and use my personal information for the purpose of this study and in the manner mentioned above.

Please note that *TEMPO* does not replace the care and advice you receive from your doctor.

Name *(please print)*: __________________________________________________________________

Click here to indicate you consent to participate in this study, submission of this online consent form replaces a signature: __________________________________________ Date: ___________________

Mailing address: _____________________________________________________________________

E-mail: ___________________________________________ Telephone (optional): ________

The best time to contact me: *Day:* _______________________ *Time:* __________________

| Would like a copy of the summary of the results to be mailed to you, on completion of this project? (Please indicate your answer with an ‘X’.) | □ Yes □ No |
| --- | --- |
| Our team has planned and ongoing studies to reduce anxiety and improve the quality of life of men with prostate cancer and their caregivers Would you like to receive information about these studies? | □ Yes □ No |

**For use by the research team**

**II. Signature of the person who obtained consent, if different from the study investigator**

I have explained the terms of the present information and consent form to the research participant and I answered all his/her questions.

______________________________________________________________________

Name and signature of the person who obtained consent Date

**III. Signature and commitment of the researcher in charge of the study**

I hereby certify that the terms of the present information and consent form were explained to the research participant, that any questions the participant had were answered and that it was clearly indicated that he/she remains free to withdraw from the study, without suffering any prejudice.

I undertake, together with the research team, to respect what was agreed upon in the information and consent form and to give a signed copy of this form to the research participant.

_______________________________________________________________________

Name and signature of the researcher in charge of the study Date
