# Supplementary material for: A study protocol for a multicenter randomized pilot trial of a dyadic, tailored, web-based, psychosocial, and physical activity self-management program (TEMPO) for men with prostate cancer and their caregivers
Source: Pilot Feasibility Stud. 2021 Mar 20;7:78. doi: 10.1186/s40814-021-00791-6 (PMC7980105; doi:10.1186/s40814-021-00791-6)
Supplement: Supplementary file 3 — Additional file 3. Sample factsheet. [file 40814_2021_791_MOESM3_ESM.pdf]

TEMPO TRUENTH A MOVEMBER INITIATIVE Prostate Cancer Canada

Knowing where to go for more support

"The extent to which a person with cancer has support and feels supported has been identified as an important factor in their adjustment to the disease."

National Breast Cancer Centre and National Cancer Control Initiative, 2003

It helps to know you will have the support you need, when you need it. While the available services and resources are extensive, they can be tricky to identify.

Suggestions for building a support team

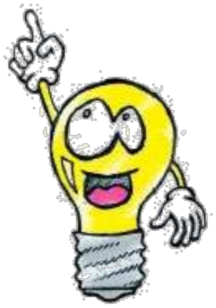

1. **Use the Canadian Cancer Society**– The Canadian Cancer Society has agencies in every province and territory. It can connect you to many useful resources according to your needs.
2. **Create your support teams** – Consider who can help address your physical, emotional, financial and social needs.

We will now explain how these suggestions can help you and provide step-by-step

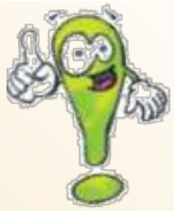

TOP TIP: Use the suggestion right for you:

- ✓ Read the description of each suggestion that follows
- ✓ Choose the strategy or strategies that you prefer
- ✓ Set your TEMPO goal(s) in Module 2 to use your preferred suggestion
- ✓ Make an action plan to help you fit your preferred suggestion into your daily life
- ✓ Set rewards for the progress you make toward achieving your goal.

guidance on how to use these suggestions.**

How can these suggestions help?

Building your support teams essentially means that you have access to supportive care that considers all aspects of your cancer experience. There are several benefits associated with this, such as:

- ✓ Reduced anxiety and depression.
- ✓ Enhanced management of physical symptoms and side effects.
- ✓ Increased understanding of cancer and its treatment.
- ✓ Greater ability to cope with treatment.
- ✓ Improved decision making and active participation in care.
- ✓ Increased satisfaction with the care provided.
- ✓ Improved quality of life.

Suggestion 1: Call the Canadian Cancer Society

A good place to start asking about resources in your area is the **Canadian Cancer Society** by calling **1-888-939-3333** (Monday to Friday), everywhere in Canada. You can get more information on their list of different services and resources for specific areas.

The Canadian Cancer Society can help you with:

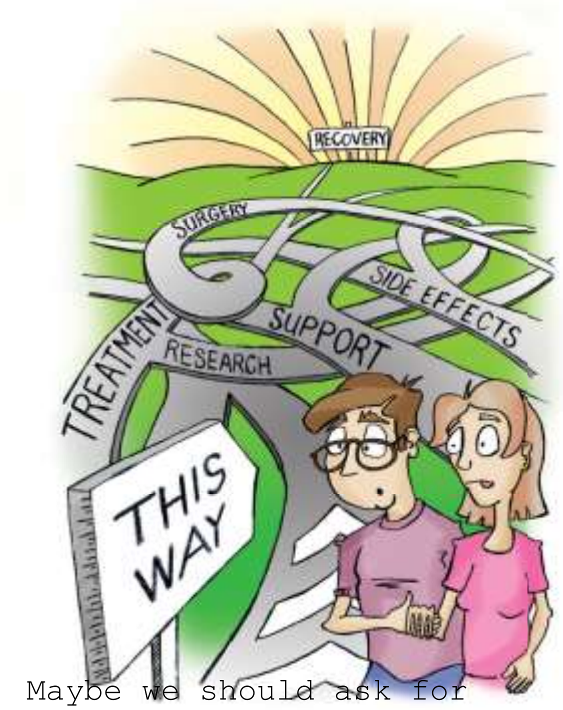

Maybe we should ask for directions?

- ✓ Cancer information service: Information about cancer, treatments, and side effects (including brochures you can request or download).
- ✓ Peer support services: information about support services in your area.
- ✓ Online community (www.CancerConnection.ca): a peer-based support service which links you to with others in similar situations.
- ✓ Information about current cancer research.
- ✓ Access to other support services in your community through their service called the "Community Services

Locator".

Suggestion 2: Create your support teams

Three main teams help you through the cancer experience. These are:

Health Care Team: Includes all the health care professionals involved in your care:

- Oncologist
- Oncologist surgeon
- Family doctor
- Oncology nurse
- Community nurse
- Social worker
- Physiotherapist
- Occupational therapist
- Psychologist/ Counsellor
- Palliative care team
- Spiritual care worker
- Pharmacist
- Dietitian

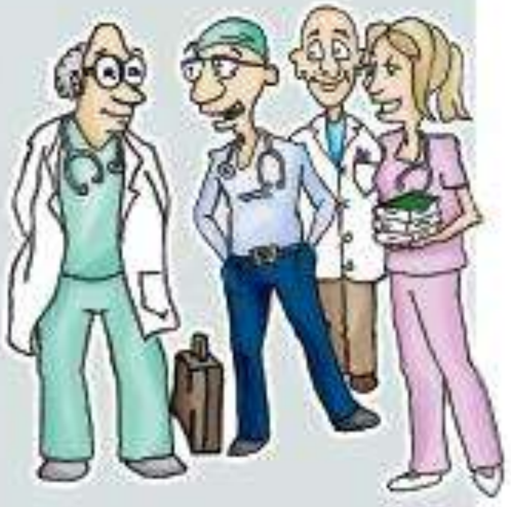

Support Services Team: Includes all the services that might be able to help you with:

- Carer respite
- Home care
- Financial services
- Counselling
- Interpreters
- Home maintenance
- Transport
- Mobility
- Legal services
- Support groups
- Housework
- Accommodation

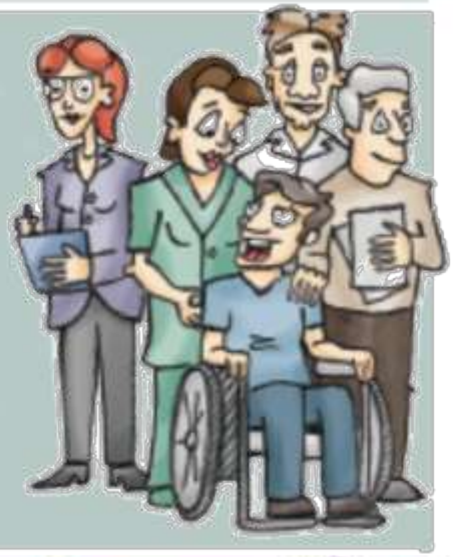

Friends and Family: Includes everyone in your informal support network who might be willing and able to provide help:

- Family members
- Friends
- Spiritual group members
- Work colleagues
- Neighbours
- Parents from your children's school

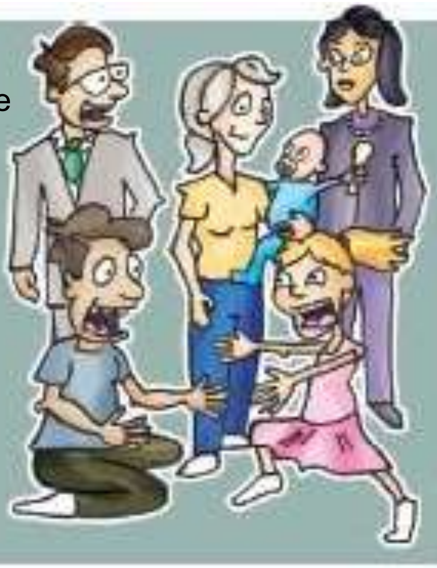

The first step in building your teams is to consider the type of support you need. Take some time to read through the table below to identify the services and assistance you need **now** and the areas in which you may need help over the next few months. We have suggested who is most likely to assist – you can add the names and contact details of the people and services that you deal with to create a handy reference guide.

Problem	Do I need help?	Who can help?	Name and contact details of who can help
Understanding treatment	NOW □ FUTURE □	Nurse, family doctor, oncologist, oncology nurse, community nurse	
Treatment side effects	NOW □ FUTURE □	Oncology nurse, family doctor, nurse, oncologist, community nurse	
Physical rehabilitation	NOW □ FUTURE □	Physiotherapist, massage therapist, occupational therapist, nurse, community nurse	
Managing medications	NOW □ FUTURE □	Family doctor, oncologist, nurse, community nurse, pharmacist	
Pain management	NOW □ FUTURE □	Nurse, community nurse, family doctor	
Follow-up nursing care (e.g. dressings)	NOW □ FUTURE □	Community nurse, nurse, family doctor	
Hygiene/ personal care (e.g. showering, wigs)	NOW □ FUTURE □	Community nurse, community service provider	
Depression, anxiety, and/or stress	NOW □ FUTURE □	Psychologist, psychiatrist, family doctor, counsellor, pivot nurse, social worker, family and friends	
Lack of emotional support	NOW □ FUTURE □	Psychologist, family doctor, counsellor, social worker family and friends, community service provider	
Sexual issues	NOW □ FUTURE □	Psychologist, counsellor, sex therapist, family doctor, nurse	
Isolation	NOW □ FUTURE □	Family and friends, social worker, support	

Problem	Do I need help?	Who can help?	Name and contact details of who can help
		groups, psychologist, counsellor	
Carer needs (e.g. training in lifting)	NOW □ FUTURE □	Social worker, community service provider	
Carer respite	NOW □ FUTURE □	Community service provider, social worker, family and friends	
Mobility	NOW □ FUTURE □	Physiotherapist, occupational therapist, community service provider	
Looking after children	NOW □ FUTURE □	Family and friends, community service provider	
Dietary changes	NOW □ FUTURE □	Dietitian	
Transport (e.g.appointments, children to school)	NOW □ FUTURE □	Community service provider, social worker, family and friends	
Domestic help, housework, and/or home maintenance	NOW □ FUTURE □	Community service provider, family and friends, social worker	
Preparing meals	NOW □ FUTURE □	Community service provider, family and friends, social worker	
Shopping	NOW □ FUTURE □	Community service provider, family and friends, social worker	
Completing forms	NOW □ FUTURE □	Family and friends, social worker	
Accommodation	NOW □ FUTURE □	Social worker, community service provider	
Crisis planning	NOW □ FUTURE □	Nurse, family doctor, social worker, family and friends	
Legal issues	NOW □ FUTURE □	Community legal centres, government offices, notary	
Other:	NOW □ FUTURE □		

TOP TIP: Most of the problems listed in the above table are addressed by factsheets in this section of the TEMPO Health Library. Review these factsheets to help you find the support you need

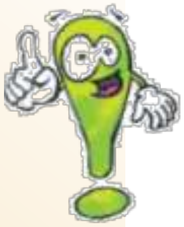

How do I build my Medical Team?

Your medical team will largely be built around you. Some members of the team will be in frequent contact (e.g. doctors). Others may only come in for a short time or you may need to ask to access to them. If you don't know who to ask about accessing additional health care services (e.g. psychologist, dietitian), a good starting point is the nurse or a social worker.

How can a social worker help?

Oncology social workers are trained to offer proper help to people with cancer and their families. Social workers can offer the following:

- ✓ Help with practical needs.
- ✓ Refer to support groups.
- ✓ Offers financial assistance and other resources such as home care and equipment, help with employer, filling out applications, money problems related to cancer, and information about health insurance.

Ask your doctor or nurse to recommend a social worker or check with the Canadian Cancer Society to pout you into contact with a social worker in your area.

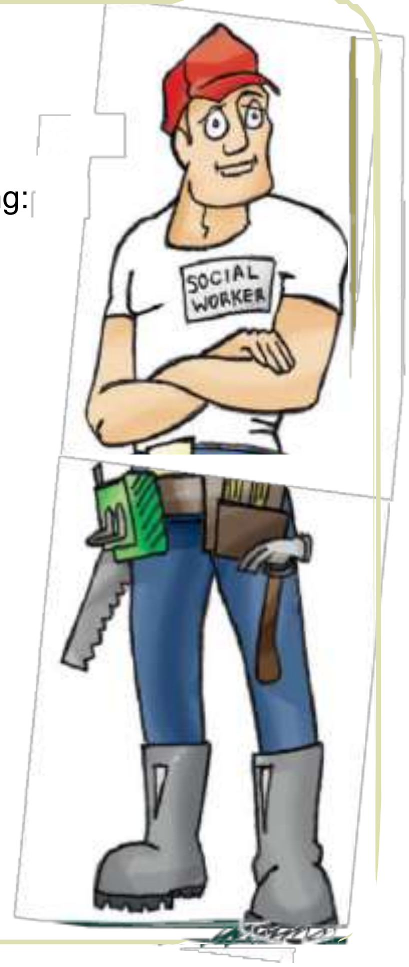

TOP TIP: Remember, you have the right to a second opinion on any medical issue or treatment decision. If you feel unable to work with a member of your health care team, you have the right to ask for someone else.

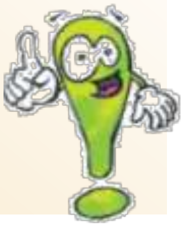

If you need help from someone on your medical team, but are not sure who or what to ask, **see the section *'Knowing the role of all of the health care professionals involved'*** in this factsheet.

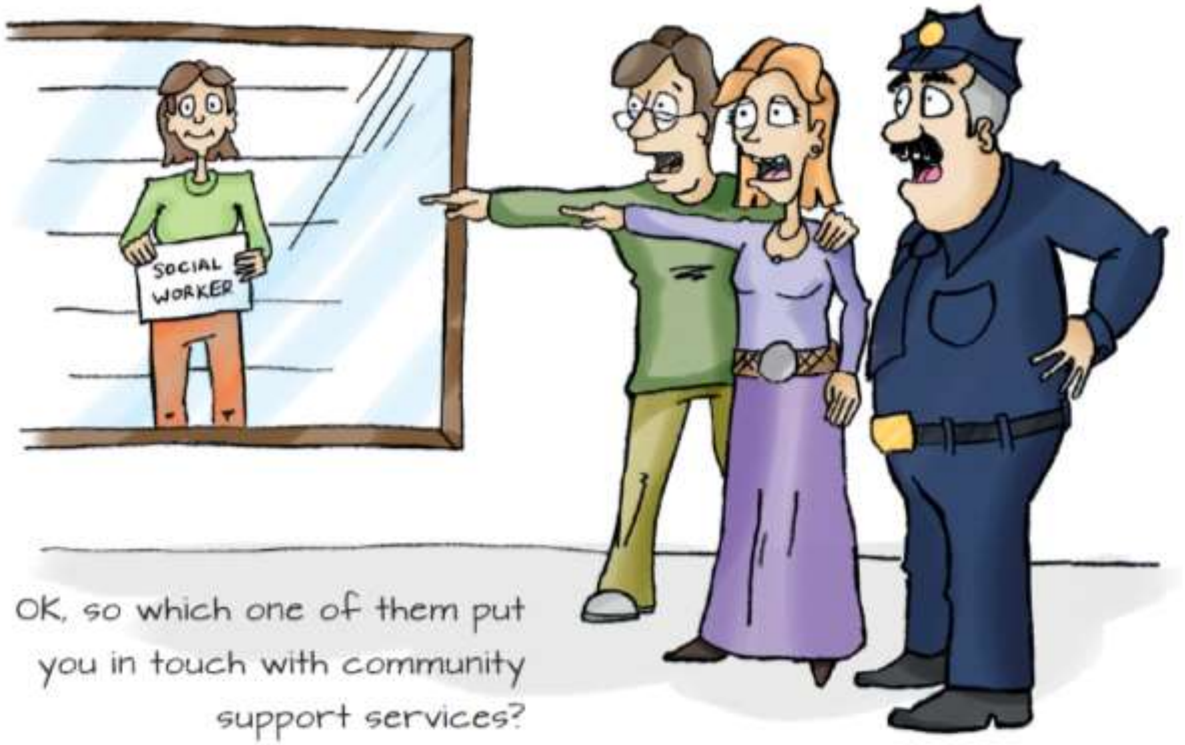

How do I build my support services team?

Once you have identified the issues you need to be addressed, you need to find out whether there are services in your area that can help.

There are many ways to find out about local services. You can:

- ✓ Ask your doctors or nurses.
- ✓ Ask your social worker.
- ✓ Ask friends and family.
- ✓ Call the Canadian Cancer Society (1-888-939-3333) and ask for the “Community Services Locator” service.
- ✓ Look through the local phone book.
- ✓ Look through the local newspaper.
- ✓ Search the Internet.
- ✓ Visit the local library.
- ✓ Contact organizations that you know – even if they do not provide the service you need, they may know who does.

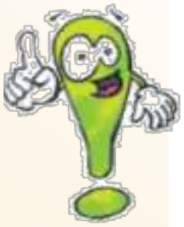

TOP TIP: The Canadian Cancer Society offers a service called the “**Community Services Locator**” which directs you to many services according to your location and community. Whether you are the person diagnosed or a caregiver, this service will help you to find information about the many services available to you.

The service locator includes as many as 4000 services specific to cancer, for example, you can get information regarding emotional support programs, homecare, treatments, and other.

You can reach this service by visiting the Canadian Cancer Society’s website: www.cancer.ca or by calling them at 1-888-939-3333.

How do I build my family and friends team?

Family and friends are a valuable source of support. Not knowing how they can help may stop some people from offering, even though they would be more than happy to do so. Unfortunately, many patients and caregivers look back and believe they took too much on themselves since they were reluctant to ask for help.

Once you have identified the help you need, consider which of your family and friends could best provide it. For example, someone with a great relationship with many of your family and friends might make an excellent contact for updates on your progress. They could then let you know who has called and you could call them back when you feel up to it.

Knowing who you can count on for what kind of help is important. Ask your family and friends to think about the help they can provide – an easy way to do so is to get them to complete a checklist like the one below.

Help I can offer	Name:
□ Mow lawns	□ Communication contact point
□ A meal prepared occasionally/nights a week/fortnight	□ A weekly phone call
	□ Research information for you
□ Help with forms / other paperwork	□ Home maintenance
□ A shoulder to cry on	□ Run errands
□ Grocery shopping	□ Gardening
□ Babysitting	□ Pick up medications
□ Respite for spouse/partner	□ Attend appointments
□ Visit you in hospital	□ Clean the house
□ A regular ride at a pre-assigned time (e.g. take children to school, take you shopping)	□ Take you out, do something social
	□ Help with washing/ironing

Adapted from National Family Caregivers Association

TOP TIP: Talk to your family and friends about the help you might need.

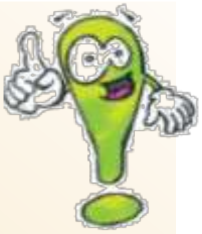

People often WANT to help but don't know how or don't want to impose. If family or friends offer to help, ask them how long they can do it for. Some people will be happy to help regularly for as long as it takes, others will only be able to help occasionally. It is good to know this up front.

TOP TIP: If you asked someone for help, be prepared for people who say 'no'. You do not need to take this personally. Some people might be dealing with their own

What others have said about using these strategies:

"Before I was diagnosed, I remember being amazed by the many services which were set up for cancer patients. It wasn't until I needed them that I realized the problem was not what was around, but where."

Dion, diagnosed with prostate cancer.

Key Points: Building a social network

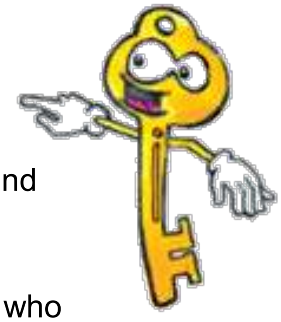

1. Your support network might include members of your medical team, services from community-based organizations, family, and friends.
2. **Determine what type of help you need** and then find out who can provide it.
3. Your social worker and the Canadian Cancer Society can help you access a variety of services.

Disclaimer

TEMPO is not a substitute for consultation with your health care professional. If you have health related questions, please call or see your health care professional. Do not disregard their professional medical advice, or delay seeking it, because of information in this program. Before you start any health treatment, always consult your health care professional. Care has been taken to ensure that the information in this program is accurate at the time of publication. All names of patients and partners or family members have been changed to protect their identity.

TEMPO is not responsible for any injury or damage to any person or property arising from, or related to, any use of the program, or to any errors or omissions. TEMPO has links to internet websites operated by third parties, and the developers of TEMPO are not responsible for the content on these sites. These links are provided to you for further information only. The inclusion of any link does not imply any endorsement of these websites by the developers of TEMPO and we are not supporting advertisements that may appear on these websites
